# State of the Oral Implantology Practice in Spain during the COVID-19 Pandemic: A Cross-Sectional Survey

**DOI:** 10.3390/ijerph20031743

**Published:** 2023-01-18

**Authors:** Ángel-Orión Salgado-Peralvo, Daniel Fernández-Cerero, Alvaro Garcia-Sanchez, Naresh Kewalramani, Eugenio Velasco-Ortega, Álvaro Jiménez-Guerra, Iván Ortiz-García, Jesús Moreno-Muñoz, Enrique Núñez-Márquez, José López-López, Loreto Monsalve-Guil

**Affiliations:** 1 Department of Dental Clinical Specialties, Faculty of Dentistry, Complutense University of Madrid, 28040 Madrid, Spain; 2 Department of Stomatology, Faculty of Dentistry, University of Seville, 41009 Seville, Spain; 3 Department of Oral Health and Diagnostic Sciences, School of Dental Medicine, University of Connecticut Health, Farmington, CT 06030, USA; 4 Department of Nursery and Stomatology, Faculty of Dentistry, Rey Juan Carlos University, 28922 Madrid, Spain; 5 Department of Odontostomatology, Faculty of Dentistry, University of Barcelona, 08907 Barcelona, Spain

**Keywords:** SARS-CoV-2, COVID-19, practice management, dental, practice patterns, dentists, dental implants, implant dentistry, surveys and questionnaires

## Abstract

At the beginning of the COVID-19 pandemic, strict measures of confinement and social distancing were taken. Dentists were considered essential personnel and their activity was restricted to emergency treatment. The present observational study aims to determine the situation of oral implantology practice in Spain during the initial period of the COVID-19 pandemic. This is a cross-sectional observational study based on the STROBE (Strengthening the Reporting of Observational Studies in Epidemiology) guidelines. An electronic survey consisting of three blocks of questions was sent to all members of the Spanish Society of Implants. The data were analyzed using descriptive analysis. A total of 237 participants (14.3%) responded to the questionnaire. The majority of participants (60.8%) only attended emergencies during the first 9 months of the pandemic. Despite this, 77.2% reported having performed dental implant surgeries and 75.5% indicated that they performed non-essential treatments. The activity was fully recovered by 64.1% of the surveyed dentists. The majority of dentists (90.7%) considered that sufficient/adequate preventive measures were carried out at their workplace, which possibly contributed to the fact that 49.3% were not afraid of becoming infected. This concern was significantly and directly proportionally associated with the age of the surveyed dentists. The oral implant practice was affected to a greater extent during the first 9 months of the pandemic, especially in urban areas, with a greater impact on the workload of professionals with less specialised training in oral implantology.

## 1. Introduction

COVID-19 (coronavirus disease 2019) is a disease caused by a virus called the Severe Acute Respiratory Syndrome-Coronavirus-2 (SARS-CoV-2) and was discovered in December 2019 in Wuhan, China. It is a highly contagious acute respiratory syndrome, which caused it to spread globally very rapidly [1], with more than 494 million cases and more than 6.17 million deaths reported by April 2022 [2].

The situation changed for the world’s population on 11 March 2020, when the World Health Organization (WHO) declared COVID-19 a pandemic [3]. To combat the spread of the disease, most countries implemented unprecedented containment measures, such as closing schools, restaurants, and shops, restricting domestic and international travel, and implementing social distancing measures, avoiding interpersonal meetings altogether [4]. In this regard, Spain declared a “State of Alarm” on 14 March 2020, prohibiting freedom of movement and imposing a blockade and, on 29 March, it was announced that all non-essential workers were ordered to confine themselves to their homes [5].

However, the closure of dental practices was not mandatory, as they were considered medical facilities [6]. Most dental professionals postponed elective dental treatments, providing only emergency care, as recommended by the General Council of Dentists of Spain [7]. These recommendations followed the guidelines of the American Dental Association (ADA) [8] in terms of what was considered to be a dental emergency. At the same time, many decided to close their dental practices and wait for the situation to improve. Dental practices had to implement costly and complicated safety measures in a short period of time, mainly by changing the way patients accessed dental care units and adjusting strategies for delivering quality care. During the first months, there was a shortage of personal protective equipment (PPE) and many dental practices struggled to have sufficient PPEs for their teams [9,10]. On 4 May 2020, restrictions limiting non-urgent treatments were lifted [5], so dental implant (DI) procedures could be performed. However, due to the shortage of PPEs and the widespread fear in both the patient population and dental professionals [11], the number of these procedures was potentially affected. For example, a longitudinal study on Australian youth observed that 36.9% of them avoided healthcare during the first year after the outbreak [12]. Similarly, a study on college students showed that 11% of the students could not get proper access to care during the COVID-19 pandemic [13]. Dentists are one of the groups most at risk of COVID-19 infection due to their proximity to the patient and the frequent generation of aerosols from dental treatments, which may have contributed to the decision to close practices and the hesitation to see non-urgent patients [14].

As health professionals, dentists were affected both in the prevention and spread of infection and in the way care was delivered. In this regard, 38% fewer patients presented for dental care at the beginning of the pandemic compared to the period before the outbreak [15]. In particular, implant dentistry was one of the most affected specialties due to the combination of surgical, prosthetic and aerosol-generating interventions [16].

To the best of our knowledge, no similar research has been conducted on Spanish dentists involved in DI procedures during COVID-19. Therefore, this observational study aimed to evaluate the current state of the practice of oral implantology 18 months after the outbreak of COVID-19 in Spain and to compare it with that of the first months of the pandemic in a population of Spanish dentists.

## 2. Materials and Methods

### 2.1. Study Design

A cross-sectional observational study was carried out following STROBE [17] (Strengthening the Reporting of Observational Studies in Epidemiology) Guidelines. It was conducted in collaboration with the Spanish Society of Implants (*Sociedad Española de Implantes*—SEI).

### 2.2. Questionnaire

For the study, a new COVID-19 questionnaire was developed to determine, the occupational consequences among dentists involved in oral implantology 18 months after the onset of the COVID-19 pandemic. The questionnaire was sent via Google Drive and was open to all respondents from December 2021 to January 2022, during which time three reminders were sent so that those who had not completed the questionnaire could do so. No exclusion criteria were applied. The response to the questionnaire was voluntary and anonymous, as it did not contain any personal information that could identify the participants and did not allow the respondent to be identified. No direct benefits were offered for participation. This study was conducted in accordance with the Declaration of Helsinki.

The adequacy of the content of the questionnaire was reviewed by experts to assess the clarity of the wording of the items before the main study. The survey was divided into three blocks of questions. The first block, consisting of nine closed questions, inquired about general data related to the surveyed professional, such as demographic data, data related to academic training in the fields of oral implantology, oral surgery, periodontology, and/or their combinations, professional data, and whether someone close to the respondent suffered serious consequences stemming from COVID-19. The second block, consisting of 11 closed questions, asked about concerns about contracting the disease and about work-related consequences and consequences related to dental clinic attendance. Finally, the third block, consisting of six questions, two of them multiple-choice, investigated preventive transmission control measures against COVID-19, as well as their acceptance by patients. All questions were compulsory, as without answering one question it was not possible to proceed to the next one. The full questionnaire is presented as Supplementary Materials (File S1).

### 2.3. Recruitment of Participants

The survey was sent to all dentists who were current members of the SEI and who had not expressed a wish not to receive e-mails (*n* = 1661) via the following link: https://docs.google.com/forms/d/1eOfaS39CMtbhA7SAtnPeOnAxBIGdK4fPzbNzIjo6e6k/edit (access from 2 December 2021 to 31 January 2022). Completion of the survey implied the participant’s consent to the collection of this information. The final sample consisted of those professionals who chose to fully respond to the survey (*n* = 237). Each respondent could answer the electronic survey once, and the options for each question, as well as the questionnaire variables, are shown in Table 1, Table 2 and Table 3.

### 2.4. Statistical Analysis

The questions were entered into Google Forms for electronic distribution and data collection. Data were exported to Microsoft Excel for cleaning and manipulation of variables for further analysis. Collected data were analysed with IBM^®^ SPSS Statistics v.26 (IBM^®^ Corp., Armonk, NY, USA), and 95% confidence intervals (CI) were applied. All descriptive variables of the subjects were determined as cross variables, and all study variables were treated qualitatively. The chi-square test was used.

### 2.5. Bias

There could be no selection bias, as the electronic survey was sent to all dentists registered in the SEI. The platform used for data collection and survey completion was set up to avoid duplicate responses. A description of the purpose of the survey was included in the survey mailing along with the link to the questionnaire and the consent form.

## 3. Results

### 3.1. Participants

The survey was responded to by a total of 237 participants; thus, the response rate was 14.3%, which was considered an appropriate number.

### 3.2. Descriptive Data

The survey was answered by 165 men (69.6%) and 72 women (30.4%). Half of the sample was composed of participants between 31–40 years of age (24.5%) and over 60 years (23.6%). In general, the respondents had a high level of academic training related to oral implantology, with almost one-third of the respondents in possession of a completed Master’s degree related to Oral Implantology (55.7%) or being in the process of completing one (5.1%), and 24.5% having completed specialized university degrees.

The practices in question are mainly private practices (78.9%), and most are not dedicated exclusively to implant dentistry (84%), as they combine it with other types of treatment. Despite this, they perform a high number of DIs per year, with 46.4% performing more than 100 DIs per year and 29.5% performing between 51 and 100 DIs per year. Only a quarter of those surveyed performed fewer than 50 DIs per year (24.1%). Concerning the number of years of experience performing these treatments, the distribution between the different groups was fairly homogeneous, with the group with 6 to 15 years of experience predominating (28.7%), followed by the group with more than 20 years of experience (26.2%) and the group with less than 5 years (26.2%) of experience. The least represented were those with 16 to 20 years of experience (19%). Some 85.2% worked at an urban level, while only 14.8% worked at a rural level.

Almost half of the participants (48.9%) do not know anyone close to them who suffered the consequences of COVID-19, and only 3% had it themselves. The other half of the respondents know co-workers and/or friends (25.7%) or a close relative (22.4%) that was infected. In rural areas, the majority of dentists (77.1%) did not know anyone close and/or known to them who had severe COVID-19 (*p* < 0.001) (Table 1).

### 3.3. Main Results

#### 3.3.1. Concern about Contracting COVID-19 and Work- and Care-Related Consequences

The majority of respondents (60.8%) only attended emergencies during the initial period of the pandemic, i.e., the first 9 months, with those aged 41–50 years attending the most emergencies (72.1%; *p* < 0.05) and those under 31 years of age attending the least (35.3%; *p* < 0.01). More than one-third of the respondents (34.6%) did not provide any care at all, and 4.6% continued with their care services without any change. Despite this, 77.2% of the dentists reported having performed DI surgeries during this period, and 75.5% performed other non-essential treatments related to Oral Implantology, such as second-stage surgeries and the placement of prostheses on DIs, among others. From the first 9 months of the pandemic onwards, virtually all participants resumed DI placement surgery (95.8%). Despite this, 0.8% of respondents continued to suspend their activity, while the rest resumed it completely (64.1%) or partially (34.6%). In particular, more than half of the professionals had fewer patients after the first 9 months of the pandemic than at the beginning of the pandemic (28.3%), significantly affecting those over 60 years of age (48.2%; *p* < 0.001), or approximately the same (30%). On the other hand, 41.8% reported having a higher volume of patients. The age group that saw the greatest increase in the number of patients after the first part of the pandemic were those aged 41–50 years (55.8%; *p* < 0.05) and, concerning the environment, 60% of those working in rural areas saw a significant increase in the number of patients after the first 9 months (*p* < 0.05).

To date, most clinics have not reduced the number of staff employed (71.7%) or the number of hours they work (20.3%). However, 8% had to lay off some of their staff or some of them left their jobs.

Half of the respondents (50.6%) were worried about contracting COVID-19 during their dental practice, mainly because of fear of infecting their family members (50%). This concern was significantly higher in the 41–50 age group (73.9%; *p* < 0.05). On the other hand, about half of the respondents were indifferent (31.6%) or not worried (17.7%) about contracting the disease. In this sense, 67.1% had a decreased level of concern after the first 9 months of the pandemic. In general, an inverse relationship was observed between age and fear of infection (*p* < 0.001), with those under 30 years of age being the least worried (82.4%; *p* < 0.05) (Table 2).

#### 3.3.2. Preventive Measures to Control Transmission of COVID-19

Most professionals (71.3%) do not inform patients of the risk of contracting COVID-19 before their dental appointments and those who do provide such information verbally (40.1%), include it in the informed consent form (39.7%), or via social networks and/or on the clinic’s website (11%).

Despite this lack of information, 96.2% of respondents claim to take specific preventive measures against SARS-CoV-2, such as monitoring body temperature (71.3%) and having patients complete an exposure questionnaire (51.1%). Overall, the percentage of dentists requesting a PCR or antigen test in the last 3–7 days before a dental appointment is very low (1.7% and 3%, respectively). Other protective measures, such as the use of PPEs, were used less than at the beginning of the pandemic (63.3%), while 33.8% used the same amount.

Half of the dentists (49.8%) used (and continue to use) additional aerosol prevention measures at the beginning of the pandemic, while 27.8% did not use them anymore. Their non-use is significantly associated with age (*p* < 0.01), as 71.4% of those over 60 years of age continued to use these measures (*p* < 0.001), while only 29.4% of those under 30 years of age did so (*p* < 0.01). A total of 22.4% did not implement specific means to reduce aerosol generation and/or control.

In general, dentists considered that adequate measures had been taken in their workplaces to prevent or reduce the risk of exposure (90.7%). On the other hand, 81% of the dentists indicated that the patients rated these measures positively as being necessary, 18.1% indicated that they were indifferent to them, and only 0.8% believed that they were unnecessary (Table 3).

## 4. Discussion

The dental profession has not had universal guidelines on how to manage COVID-19, despite being perceived as having an extremely high risk of exposure among all professionals [18,19,20]. In Spain, especially during the peak of the pandemic, many patients were unable to receive scheduled treatment because activity was restricted to emergencies by government mandate [21]. Private and public dentistry were no exception, and only dental emergencies were treated. According to the ADA [8], urgent procedures related to oral implantology included: (1) uncontrolled bleeding, (2) stitch removal, and/or (3) prosthesis adjustment when it impeded masticatory function. Moreover, the American Association of Implant Dentistry (AAIP) [16] released a white paper discussing the guidelines for DI-related treatments during the COVID-19 pandemic. They included loss or fracture of an existing dental restoration, infection of the peri-implant tissues, and DI mobility. The rationale is that these scenarios can lead to the loss of DI(s), causing functional, financial and emotional costs. In the present study, 60.8% of the respondents reported having attended only DI emergencies, which is significantly higher than those reported by an international survey of 2318 Spanish dentists, of whom only 39.5% reported having done so [22]. In other countries, these figures were lower [23]. Interestingly, 14.2% of Australian dentists who only treated emergencies avoided telling others for fear of receiving negative reactions, and 10% felt that family or friends avoided contact with them [24].

As might be expected, the more treatments performed, the higher the risk of complications. Thus, 65.5% of professionals performing more than 100 DIs per year had DI complications in the first 9 months (*p* < 0.05) compared to 33.3% of those performing up to 50 DIs per year (*p* < 0.00001). These figures are also associated with the fact that 88.2% (*p* < 0.001) of professionals performing more than 100 DIs per year also performed these surgeries during the initial period of the pandemic, which was against the recommendations at the time. Moreover, 75.5% of all practitioners surveyed reported performing non-essential treatments during this period.

The bulk of DI emergencies were handled by practitioners aged 41–50 years, which was related to this same age range having a significantly higher concern about infecting their family members (73.9%; *p* < 0.05). Overall, however, concern about infection was low, with half of the respondents (50.6%) being concerned, similar to those reported in other surveys conducted in Australia (58.7%) [24] or Italy (50.5%) [25]. It has also been observed that such concern is inversely proportional to age (*p* < 0.001), as was also the case in another study conducted in Texas (USA), where the cut-off age was 55 years (odds ratio = 1.55; *p* = 0.043) [26]. These data were probably motivated by the high incidence and mortality of COVID-19 in elderly people (with pre-existing health conditions), with a median age ranging from 51 [27] to 78 [28] years, and the fear that it generates in dentists over 60 years. On the other hand, a higher proportion of young patients did not know anyone close to them who had the virus, especially in the 31–40 years age range (60.3%; *p* < 0.05), which may have led them to justify the risk of being infected and to be a route of transmission of the disease to their environment.

The pandemic has been a difficult period at the health and emotional level, as indicated by some surveys which put the percentage of professionals who have been psychologically affected at more than 80%. More specifically, 67.6% of maxillofacial surgeons have shown psychological distress, and 78.2% of general dentists have shown it. It also increased directly proportional to an increase in knowledge about COVID-19 [29]. In neighboring countries such as Italy, they observed a significant increase in mortality rates for dentists and physicians in 2020 and 2021 [30,31]. These emotional disturbances were also due to uncertainty about the consequences for their professional future, and about the end of the pandemic restrictions [25]. Other serious consequences were financial in nature [32]. A survey conducted in Europe showed that, before the pandemic, most dentists saw 6–15 patients per day, whereas, during the critical period of the pandemic, this number was 0–5 patients per week. Furthermore, three out of 4 dentists in Italy confessed that the pandemic period affected them in an extremely negative way [25]. Fortunately, 71.7% of dental clinics did not lay off workers. An important role in this was played by the Record of temporary employment regulation (in Spanish, Expedientes de Regulación Temporal de Empleo, ERTEs), a labour measure that enables the company to reduce or suspend employment contracts. As of 31 December 2020, this measure affected 755,613 workers in Spain [33]. In Italy, where economic measures were also developed to mitigate the impact of the health crisis, it resulted in 45% of dentists attending emergencies by themselves, while 55% were aided by a single assistant [25]. In Spain, considering that 90% of dentists are self-employed [34], these figures could have been higher given the impact of ERTEs.

The profile of professionals who recovered their clinical practice earlier in the pandemic were those who completed a Master’s Degree related to Oral Implantology or Oral Surgery, Periodontics and/or combinations (72%; *p* < 0.01), perhaps because 83.3% performed DIs during the initial period of the pandemic (*p* < 0.05). The performance of such non-essential treatments was also associated with private practice exclusively (80.2%; *p* <0.01), possibly to mitigate the economic consequences. On the other hand, professionals who did not know anyone close to them who suffered from the severe medical consequences of COVID-19 experienced the greatest increase in patients after the worst part of the pandemic was behind them (49.1%; *p* < 0.05), probably because they normalised this situation earlier. This can be seen, among other data, from the fact that 71.6% of these professionals used significantly fewer PPEs 9 months into the pandemic compared to the beginning of the pandemic (*p* < 0.01). In contrast, the practitioners who continue to have fewer patients are those without specific postgraduate training, suggesting that those less prepared may have been forced to close their dental practices or lose their jobs. The highest percentage of this group is represented by those over 60 years of age (7.1%; *p* > 0.05), although this did not translate into a higher number of retirements (*n* = 1; 0.4%).

Taking into account that dental professionals (dentists, dental hygienists and dental assistants) were considered at the highest risk category for SARS-CoV-2 exposure, according to USA’s Occupational Safety and Health Administration Agency [20], only 3% of dentists seriously suffered the consequences of COVID-19, which is in line with the data presented by the COVIDental Collaboration Group [22], in which the positivity rate of Spanish dentists was 3.2%, compared to 4.1% in the general Spanish population. Data from similar studies carried out in Europe and the USA have also shown low prevalence and positivity rates among dental professionals, suggesting that current COVID-19 transmission control recommendations may be adequate [25,35,36,37]. In the same vein, since the HIV/AIDS onset and the increased awareness of hepatitis B and C in the 1990s, the dental profession adopted strict infection control measures, hence dentists have a culture of infection control [38]. Thus, just as any patient is treated as if they were HIV-positive, they were now treated as if they were COVID-19 positive.

Implementation of effective infection control measures was recommended to prevent nosocomial coronavirus infection [39]. In this regard, the ordinal multinomial logistic regression showed that only the use of N95/FFP2 masks significantly reduced the probability of reporting signs/symptoms of COVID-19 [40]. In this regard, it has been speculated that dental professionals who use N95 masks at work are more likely to use them in their daily lives as well, reducing the risk of community transmission. These lower transmission rates in dental personnel have been directly associated with Gross National Income per capita (GNI), with lower rates observed in countries with higher GNIs. In this regard, in eight of the countries analysed by a worldwide survey (Pakistan, Russia, Saudi Arabia, Tunisia, Netherlands, Lithuania, Malaysia and China), the dental professionals’ self-reported COVID rate was higher than that of the general population [22].

At the beginning of the pandemic, there were many challenges to finding PPEs, including delivery times [25]; however, more than 90% of dentists consider that adequate measures were taken in their workplace to prevent the risk of exposure to COVID-19, which was not the case in other countries such as Turkey, where only 12.36% of dentists had access to N95 masks and other means of protection [29]. In addition, physicians and hospitals struggled with the same shortages, causing potential collateral damage. Numerous studies showed that due the collapse of hospitals, there was a global reduction in hospital admissions for all cardiovascular diseases and cervical cancer [41,42]. Nevertheless, physician engagement was significantly higher than in the pre-pandemic period, as they were attempting to mitigate the impact of the COVID-19 in hospitals [43]. Furthermore, it has been identified that conducting routine clinical audits helps to create recommendations during epidemics and pandemics, and potentially expands the scope of practice for healthcare providers [44].

It has been confirmed that COVID-19 is transmitted through human contact and in the form of droplets, but airborne transmission has not been ruled out [15,45]. The use of a rubber dams and strong saliva ejectors minimizes the production of aerosols during dental treatments. Other tools, such as face shields/goggles also help to avoid blood, saliva and water splashes [46]. In the present study, a very high percentage of dentists used such measures (77.6%), however, 27.8% stopped using them after 9 months.

Finally, a very high percentage of dentists (81%) stated that patients considered the preventive measures necessary. It is possible that this confidence may have made patients less afraid of becoming infected, as indicated by a survey conducted in Italy, with a mean concern score of 1.06 points (meaning 0—“no concern”, and 4—“extreme concern”). Similarly, the vast majority of patients in other countries understood the reasons for the ceasing or reduction of clinical care [25]. Some authors suggest that dentists and oral surgeons could integrate telehealth into their clinical practices in order to carry out pre-and post-operative consultations and follow-ups, thus reducing patient transit and, with it, possible transmission. These measures have been accepted positively by patients, the government and healthcare providers in the U.S. [47].

### Limitations

There are three major limitations in this study that could be addressed in future research. First, as this is a survey-based study, it is not possible to establish with certainty the veracity of the answers provided by the participants. Furthermore, this survey includes the subjective reports of respondents. Second, the number of questions is limited. Third, the differences between the various surveys published so far make it difficult to compare the data provided by the present study. These limitations were not accidentally identified during or after the survey but were known to the authors before the research began.

## 5. Conclusions

COVID-19 had a significant impact on oral healthcare in Spain. Despite this, Spanish dentists adapted adequately to the change in healthcare practices and implemented sufficient measures to decrease transmission. The need for specific training in oral implantology is highlighted by periods of upheaval such as the one we experienced, given that the best-qualified professionals were the ones who recovered their clinical practices first. Our results provide a clear picture of what Spanish dentists dedicated to implant dentistry experienced during the COVID-19 pandemic and will be useful in the management of emerging outbreaks in the future.

## Figures and Tables

**Table 1:** Demographic and professional characteristics of the study sample.

Variable	Specifications	No.^1^	% ^2^	95% CI ^3^
Gender	Female	72	30.4	25.0–35.8
	Male	165	69.6	64.2–75.0
Age (years)	≤30	34	14.3	10.2–18.4
	31–40	58	24.5	19.4–29.6
	41–50	43	18.1	13.6–22.6
	51–60	46	19.4	14.7–24.1
	>60	56	23.6	18.6–28.6
Experience with DIs ^4^ (in years)	≤5	62	26.2	21.0–31.4
	6–15	68	28.7	23.4–34.0
	16–20	45	19.0	14.4–23.6
	>20	62	26.2	21.0–31.4
Exclusive clinical practice in DI treatments	Yes	38	16.0	11.7–20.3
	No	199	84.0	79.7–88.3
Main number of DIs placed per year	<50	57	24.1	19.1–29.1
	51–100	70	29.5	24.1–34.9
	>100	110	46.4	40.5–52.3
DI education	Master´s degree	132	55.7	49.8–61.6
	Master´s degree students	12	5.1	2.5–7.7
	University specialist degree	58	24.5	19.4–29.6
	Postgraduate certificates (clinical stays, courses of commercial firms, etc.)	27	11.4	7.7–15.1
	None of the previous ones	8	3.4	1.3–5.5
Daily main practice	Private practice (only)	187	78.9	74.1–83.7
	Hospital/Multi-specialty clinic	31	13.1	9.1–17.1
	University lecturer	10	4.2	1.8–6.6
	Master’s degree student in Oral Surgery/Periodontics and Implantology	9	3.8	1.5–6.1
	Not currently practising dentistry	0	0.0	-
Working habitat	Rural	35	14.8	10.6–19.0
	Urban	202	85.2	81.0–89.4
Someone close to you was severely affected by COVID-19	A close relative	53	22.4	17.5–27.3
	Myself	7	3.0	1.0–5.0
	Co-workers and/or friends	61	25.7	20.5–30.9
	No one close and/or known	116	48.9	43.0–54.8

^1^ No., simple size; ^2^ %, percentage; ^3^ CI, confidence interval; ^4^ DIs, dental implants.

**Table 2:** Work-related and care-related consequences.

Variable	Specifications	No.^1^	% ^2^	95% CI ^3^
Initial response to the COVID-19 pandemic	Total closure	82	34.6	29.0–40.2
	Emergency treatment only	144	60.8	55.0–66.6
	No changes	11	4.6	2.1–7.1
Your practice has recovered since the COVID-19 pandemic.	Fully recovered	152	64.1	58.4–69.8
	Partially recovered	82	34.6	29.0–40.2
	Still suspended	2	0.8	0.0–1.9
	Retired during the pandemic	1	0.4	0.0–1.1
Concern about contracting COVID-19 in practice as a dentist	I do care	120	50.6	44.7–56.5
	Indifferent	75	31,6	26.1–37.1
	Not concerned	42	17.7	13.2–22.2
Major concern	My health	32	23.5	16.7–30.3
	My family’s health	68	50.0	41.9–58.1
	Infect patients or colleagues	19	14.0	8.4–19.6
	Other reasons	17	12.5	7.2–17.8
Change in concern about contracting COVID-19	Yes, it has gone down	159	67.1	61.6–72.6
	It has stayed the same	71	30.0	24.6–35.4
	No, it has increased	7	3.0	1.0–5.0
It has affected the staff of your centre	The number of hours has been reduced	48	20.3	15.6–25.0
	Staff laid off or left the job	19	8.0	4.8–11.2
	No reduction	170	71.7	66.4–77.0
Change in patients between the last 9 mo ^5^ and the first 9 mo of the pandemic	Fewer patients	67	28.3	23.0–33.6
	Approximately the same	71	30.0	24.6–35.4
	More patients	99	41.8	36.0–47.6
Emergency patient (implantology or implant-prosthesis) in the first 9 mo of the pandemic	Yes	138	58.2	52.4–64.0
	No	99	41.8	36.0–47.6
DI ^4^ surgery during the first 9 mo of the pandemic	Yes	183	77.2	72.3–82.1
	No	54	22.8	17.9–27.7
DI surgery during the last 9 mo	Yes	227	95.8	93.4–98.2
	No	10	4.2	1.8–6.6
Non-essential DI treatments the first 9 mo of the pandemic	Yes	179	75.5	70.4–80.6
	No	58	24.5	19.4–29.6

^1^ No., simple size; ^2^ %, percentage; ^3^ CI, confidence interval; ^4^ DIs, dental implants; ^5^ mo., month(s).

**Table 3:** Preventive transmission control measures against COVID-19.

Variable	Subvariable	Specifications	No.^1^	% ^2^	95% CI ^3^
Informing patients of the risk of contracting COVID-19 prior to their dental appointments	Verbally	Yes	95	40.1	34.3–45.9
		No	142	59.9	54.1–65.7
	Included in the informed consent	Yes	94	39.7	33.9–45.5
		No	143	60.3	54.5–66.1
	Via social media and/or website	Yes	26	11.0	7.3–14.7
		No	211	89.0	85.3–92.7
	No such information is given	Yes	68	28.7	23.4–34.0
		No	169	71.3	66.0–76.6
Preventive measures implemented before an appointment	Body temperature measurement	Yes	169	71.3	66.0–76.6
		No	68	28.7	23.4–34.0
	Recent exposure risk questionnaire	Yes	121	51.1	45.2–57.0
		No	116	48.9	43.0–54.8
	PCR ^4^ test in the last 3–7 days	Yes	4	1.7	0.2–3.2
		No	233	98.3	96.8–99.8
	Antigen test in the last 3–7 days	Yes	7	3.0	1.0–5.0
		No	230	97.0	95.0–99.0
	Other measures	Yes	88	37.1	31.4–42.8
		No	149	62.9	57.2–68.6
	Same measures as before the pandemic	Yes	9	3.8	1.5–6.1
		No	228	96.2	93.9–98.5
Patients’ general opinion on preventive measures		Necessary	192	81.0	76.4–85.6
		Indifferent	43	18.1	13.6–22.6
		Not necessary	2	0.8	0.0–1.9
My workplace has taken appropriate actions to prevent the risk of exposure to COVID-19		Yes	215	90.7	87.3–94.1
		Not sure	20	8.5	5.1–11.7
		No	2	0.8	0.0–1.9
Variation in PPE ^5^ use between the last 9 mo ^6^ and the first 9 mo of pandemic		They are now less used	150	63.3	57.6–69.0
		The same are used	80	33.8	28.2–39.4
		They are now more widely used	7	3.0	1.0–5.0
Additional aerosol prevention measures at the start of the pandemic		Yes, and we still use them	118	49.8	43.9–55.7
		Yes, but they are no longer used	66	27.8	22.5–33.1
		No	53	22.4	17.5–27.3

^1^ No., simple size; ^2^ %, percentage; ^3^ CI, confidence interval; ^4^ PCR, polymerase chain reaction; ^5^ PPE, personal protective equipment; ^6^ mo, month(s).

## Data Availability

The data are available in a publicly accessible repository.

## Supplementary Materials

The following supporting information can be downloaded at: https://www.mdpi.com/article/10.3390/ijerph20031743/s1, File S1: Full questionnaire.
