# Supplementary material for: State of the Oral Implantology Practice in Spain during the COVID-19 Pandemic: A Cross-Sectional Survey
Source: Int J Environ Res Public Health. 2023 Jan 18;20(3):1743. doi: 10.3390/ijerph20031743 (PMC9914189; doi:10.3390/ijerph20031743)
Supplement: Supplementary file 1 [file ijerph-20-01743-s001.zip › ijerph-1979019-supplementary.pdf]

File S1. Full questionnaire.

Block I: General variables related to the professional surveyed:

1. Sex:
   a) Female
   b) Male
2. Select your age group:
   a) ≤ 30 years old
   b) 31 – 40 years old
   c) 41 – 50 years old
   d) 51 – 60 years old
   e) > 60 years old
3. Experience with dental implants (DIs) (in years):
   a) ≤ 5 years
   b) 6 – 15 years
   c) 16 – 20 years
   d) > 20 years
4. Are you an exclusive practitioner in your clinical practice in Oral Implantology?
   a) Yes.
   b) No.
5. Average number of DIs placed per year:
   a) < 50 DIs.
   b) 50 – 100 DIs.
   c) > 100 DIs.
6. What is your education in Implant Dentistry?
   a) Master's degree in Oral Surgery/Periodontics and Implantology.
   b) Master's student in Oral Surgery/Periodontics and Implant Dentistry.
   c) University Specialist Degree in Oral Surgery/Periodontics and Implant Dentistry.
   d) Postgraduate certificates in Oral Implantology (clinical stays, courses of commercial firms, etc.).
   e) None of the previous ones.
7. Which of the following answers represents your usual/fundamental daily practice?
   a) Private practice (only).
   b) Hospital/Multi-speciality clinic.
   c) University lecturer.
   d) Master's degree student in Oral Surgery/Periodontics and Implantology.
   e) Not currently practising dentistry
8. How would you classify the area where you practice dentistry?
   a) Rural.
   b) Urban.
9. Did anyone close to you suffer the consequences of COVID-19 severely?
   a) Yes, a close relative.
   b) Myself.
   c) Yes, co-workers and/or friends.
   d) No one close and/or known.

Block II: Variables related to concerns about COVID-19 and the work- and care-related consequences.

1. What was the initial response to the COVID-19 pandemic?
   a) Total closure.
   b) Emergency treatment only.
   c) No changes.

2. Has your practice recovered after the onset of the COVID-19 pandemic?
   a) Fully recovered
   b) Partially recovered
   c) Still suspended
   d) I retired during the pandemic
3. Are you concerned about being infected with COVID-19 during your practice as a dentist?
   a) Yes, I am concerned
   b) I am neither worried nor not worried at all
   c) No, I am not worried.
4. If you are concerned, what is the main reason?
   a) I am worried about my health
   b) I am worried about my family's health.
   c) I am worried about transmitting the virus to my patients or colleagues.
   d) Other reasons
5. Has your concern about contracting COVID-19 decreased since the pandemic began?
   a) Yes, it has gone down.
   b) It has stayed the same.
   c) No, I has increased.
6. Has COVID-19 affected workforce retention at your workplace (dentists, hygienists, assistants, etc.)?
   a) Yes, the number of staff in the clinic has been reduced (reduction of hours), but no one has been laid off.
   b) Yes, workers have been made redundant or have left the job.
   c) No, there has been no reduction in staff.
7. In the last 9 months, what has been the number of DI patients per month compared to the first 9 months (March to November 2020) of the pandemic?
   a) Fewer patients.
   b) Approximately the same.
   c) More patients.
8. Did you have any emergency DI or DI-prosthetic patients during the first 9 months of the pandemic?
   a) Yes
   b) No
9. Did you perform any DI surgery during the first 9 months of the pandemic?
   a) Yes
   b) No
10. Have you performed any DI surgery in the last 9 months?
   a) Yes
   b) No
11. During the first 9 months of the pandemic, did you perform any non-essential DI treatments (i.e., procedures that did not involve removing stitches, placing prostheses that were already completed, etc.)?
   a) Yes
   b) No

Block III: Variables related to preventive transmission control measures against COVID-19.

1. Are you informing patients of the risk of COVID-19 before their dental appointments? (multiple choice):
   a) Yes, verbally.
   b) Yes, it is included in the consent form.
   c) Via social media and/or website.

d) No.

2. What kind of preventive measures are you implementing before an appointment? (multiple choice):
   a) Body temperature measurement.
   b) Recent exposure risk questionnaire.
   c) Results of a PCR test within the last 3 – 7 days.
   d) Results of an antigen test within the last 3 – 7 days.
   e) Other measures.
   f) No measures, i.e., we treat the patient as we did before the pandemic.
3. What is the general opinion of patients about preventive measures?
   a) They consider them necessary.
   b) They do not consider them necessary or unnecessary.
   c) They do not consider them necessary.
4. Do I think that my workplace has taken adequate actions to prevent the risk of exposure to COVID-19?
   a) Yes.
   b) Not sure.
   c) No.
5. In the last 9 months, regarding the use of PPE (Personal Protective Equipment):
   a) I use less PPE compared to the first 9 months of the pandemic.
   b) I use the same PPE compared to the first 9 months of the pandemic.
   c) I use more PPE compared to the first 9 months of the pandemic.
6. Did your workplace introduce additional aerosol prevention measures when the pandemic started (e.g., more hoovers, negative pressure rooms, etc.)?
   a) Yes, and we still use them
   b) Yes, but we are no longer using them.
   c) No.
